# The Prevalence of Restless Legs Syndrome in Patients with Migraine: A Systematic Review and Meta-Analysis

**DOI:** 10.1155/2020/2763808

**Published:** 2020-08-29

**Authors:** Hooman Ghasemi, Behnam Khaledi-Paveh, Alireza Abdi, Rostam Jalali, Nader Salari, Aliakbar Vaisi-Raygani, Masoud Mohammadi

**Affiliations:** ^1^Department of Nursing, School of Nursing and Midwifery, Kermanshah University of Medical Sciences, Kermanshah, Iran; ^2^Sleep Disorders Research Center, Kermanshah University of Medical Sciences, Kermanshah, Iran

## Abstract

**Background:**

Migraine is a severe and debilitating neurologic disorder and is claimed to be the sixth disabling illness in the world. This study aimed to determine the overall prevalence of restless legs syndrome (RLS) in patients with migraine through a systematic review and meta-analysis.

**Methods:**

To identify and select related studies, the Scopus, ScienceDirect, Embase, SID, IranDoc, Web of Science, Knowledge Network System Medline (PubMed), and Google Scholar databases were searched. All related cross-sectional studies, published in English or Persian language between January 2000 and December 2019 and using the keywords such as migraine, restless leg syndrome, sleep disorder, RLS, and migraine disorder, were collected. The heterogeneity of the studies was assessed using the *I*^2^ index, and the data analysis was performed using the Comprehensive Meta-Analysis software.

**Results:**

Analysis was conducted on the reported results of the final 12 articles with the total sample size of 15196. The overall prevalence of RLS in patients with migraine was 16.3% (95% CI: 12.6–20.8%). The prevalence of RLS migraine patients decreased with increasing the sample size, and the prevalence of RLS migraine patients increased with increasing the research year, which was statistically significant (*P* < 0.05).

**Conclusion:**

This study highlights that RLS is high in patients with migraine, and therefore, the clinicians should be aware of its incidence and take preventive measures.

## 1. Background

Migraine is a severe and debilitating neurologic disorder which is realised with local throbbing sensation in the head. It is a type of disturbance in the subcortical aminergic sensory modulatory systems [1–3]. In other words, the disorder is a type of brain disturbance of the subcortical aminergic sensory modulatory systems [4]. Although migraine is widespread, the causes of the disorder are not well understood, and in fact, the triggers of migraine vary from a person to another. Nevertheless, many factors such as food, barometric pressure changes, and stress can cause migraines [5]. Migraine affects the quality of life and results in healthcare costs for the patients [6].

The prevalence of migraine has been reported to be ranging from 10 to 18% in some studies. The estimated prevalence of migraine is 14.9% in European countries. In addition, migraine is reported more commonly in women than men [7–9].

Restless legs syndrome (RLS) is a movement disorder in which there is a feeling of distress and a tendency to move the legs, and in some cases the arms and the neck [10]. Although the cause of RLS in unknown, it is thought that iron deficiency in brain causes primary RLS, whereas kidney failure, pregnancy, anemia, and genetics are known to be associated with secondary RLS [11]. It occurs most often at nights, and creates sleep disturbances [12].

Migraine and RLS put a heavy burden on society [13]. These two disorders have a high impact on the quality of life. Moreover, the prevalence of RLS is higher in patients with migraine than in a healthy population [14]. Some existing studies have reported an unalike prevalence of RLS in patients with migraine. Therefore, since intervention studies on reducing the incidence of RLS in patients with migraine require accurate and consistent information to prevent the complications of RLS, an overall prevalence of RLS in migraine patients has not been reported. Therefore, this piece of research attempts to answer the following research question: what is the overall prevalence of RLS in patients with migraine?

## 2. Methods

### 2.1. Search Method

Multiple databases, including ScienceDirect, Embase, Scopus, SID, IranDoc, Medline (PubMed), and Google Scholar were searched. All related cross-sectional studies published in English or Persian language, between January 2000 and December 2019, were identified and selected. The keywords used in the search process were migraine, migraineurs, restless legs syndrome, sleep disorder, RLS, and migraine disorder. Review papers, case-control, cohort, and intervention studies were excluded from the list of articles.

Moreover, the Google Scholar search engine was searched using both Persian and English keywords. AND and OR operators were added to the keywords combinations with a view to provide a more comprehensive access to all articles. For instance, the OR operator was used to examine the common names for disorders such as (migraine OR migraine headache), (sleep disorder OR sleep wake disorder), and (restless legs syndrome OR RLS), whereas, the AND operator was added in between the keywords (AND) considering the Medical Subject Headings (MeSH).

### 2.2. Evaluation of Articles

After collecting the relevant articles, a list of abstracts was prepared. The author(s) and journal names were redacted, yet allowing reviewers to select the full-text articles. Each article was independently examined by two reviewers, and if an article was rejected, the reason for this was mentioned. In the case of disagreement between the two reviewers, the article was then reviewed by a third reviewer. Furthermore, to ensure the comprehensiveness of the search process, the Google Scholar search engine was searched to identify any related grey literature.

**Figure 1:**
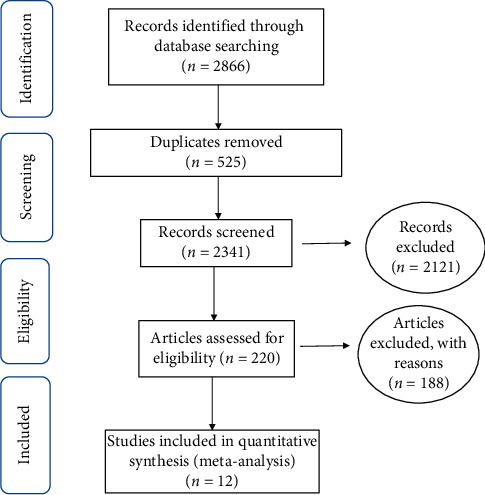
The flowchart of the stages of studies including in the systematic review and meta-analysis (PRISMA 2009).

The search process was conducted in accordance with the four-step PRISMA 2009 guidelines. The steps include article identification, screening, eligibility evaluation, and finally including the final studies for meta-analysis (Figure 1).

### 2.3. Quality Assessment and Statistical Analysis

The STROBE checklist was used to assess the quality of the studies. The total number of scores that can be obtained using the checklist is 32. Articles with a score below 14 were considered as low-quality and were, therefore, excluded from the study.

In each study, the prevalence of RLS in patients with migraine was reported. The heterogeneity of the studies was assessed using the *I*^2^ test. Data were analyzed using the Comprehensive Meta-Analysis software (Biostat, Englewood, NJ, USA version 3). The possibility of publication bias was assessed using the funnel plots and Egger's test with the significance level of 0.05. Moreover, in order to investigate the impacts of other potential factors on the heterogeneity of studies, the meta-regression test was used in the two factors of “sample size” and “year of study.”

## 3. Results

Initially, a total of 2,866 articles were identified, of which 254 articles were obtained from IranDoc, 4 articles from SID, 402 articles from Medline (PubMed), 242 articles from ScienceDirect, 378 articles from Scopus, 526 articles from Embase, 266 articles from Web of Science, and 756 articles were obtained from Google Scholar.

During the primary reviews, 525 duplicate articles were omitted; 2,121 articles were removed during the secondary review since they were unrelated to the study subject. Additionally, 188 articles were excluded due to either the lack of access to the abstract and the original article or their low-quality, and ultimately 12 articles were included in the meta-analysis (Table 1).

### 3.1. Investigation of Heterogeneity and Publication Bias

**Figure 2:**
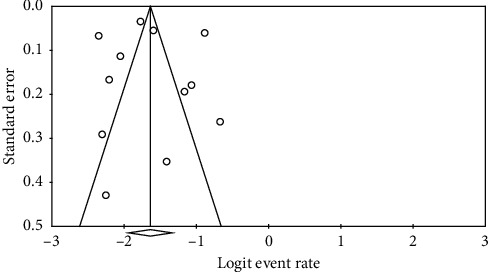
Funnel plot of standard error by logit event rate.

The heterogeneity of the studies was evaluated using the *I*^2^ test; *I*^2^ = 96.8% was obtained, indicating a high heterogeneity among the included studies. Therefore, the random effects model was used to combine the results of the studies together. Furthermore, the possibility of publication bias in the studies was assessed using Egger's test (Figure 2). The publication bias was not statistically significant (*P*=0.867).

The total sample size from all collected studies was 15,196. Based on the meta-analysis performed in this study, the overall prevalence of RLS in patients with migraine was 16.3% (95% CI: 12.6–20.8%)

**Figure 3:**
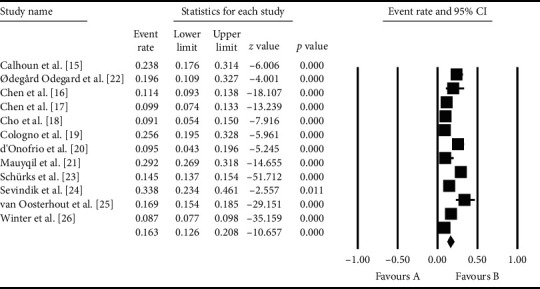
Meta-analysis.

The prevalence of RLS in patient with migraine was higher in patients ≥65 years of age in Turkey (33.8%; 95% CI: 23.4–64.1%) and, however, was lower (8.7%; 95% CI: 7.7–9.8%) in France (Figure 3). As illustrated in Figure 3, the prevalence of RLS was based on the random effects model in which the black square is the prevalence rate and the length of the line on which the square is located denotes the confidence interval of 95% for each study; the overall prevalence for the total studies is shown by the diamond shape.

**Figure 4:**
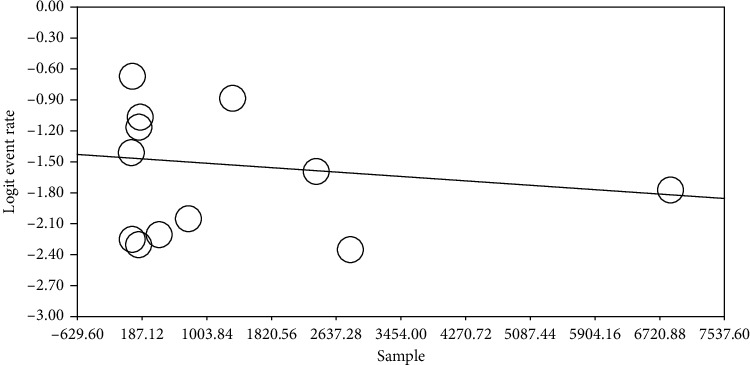
Regression of sample on logit event rate.

**Figure 5:**
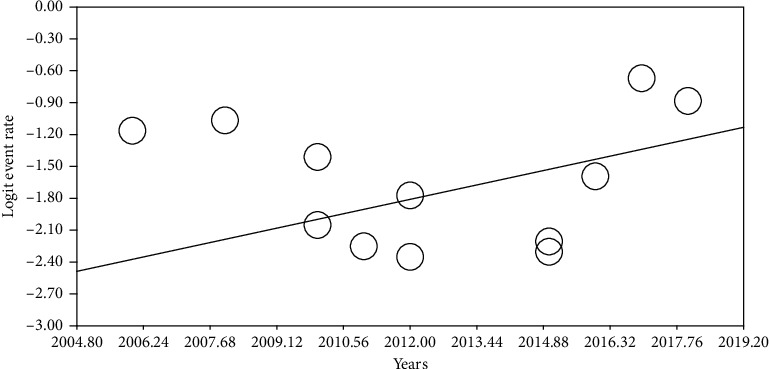
Regression of years on logit event rate.

In order to examine the impacts of potential factors on heterogeneity of RLS in patients with migraine, a meta-regression analysis was conducted using the two factors of “sample size” and “year of study” (Figures 4 and 5). According to Figure 4, as sample size increases, the prevalence of restless legs syndrome in patients with migraine decreases, and this was statistically significant (*P* < 0.05). It is also reported in Figure 5 that with increasing the year of study, the prevalence of restless legs syndrome in patients with migraine increases, which was also statistically significant (*P* < 0.05). To explain this, it can be argued that with increasing the sample size and therefore more healthy people entering the study, the incidence of cases in the study of prevalence decreases, and also with the updating of studies and increase in diagnostic methods and their improvement, the prevalence increases.

## 4. Discussion

Epidemiological studies have investigated a variety of migraine-related disorders such as anxiety, depression, and sleep problems among patients [27]. In fact, the World Health Organization (WHO) stated that migraine is a major cause of debility in the general population [18].

Restless legs syndrome/Willis–Ekbom disorder (RLS/WED) is a movement disorder that most commonly affects the legs and can cause severe sleep disturbance [28]. According to a study conducted by Cho et al., the prevalence of this disorder was estimated to be 2–15%. Moreover, the prevalence increases with age. The International Restless Legs Syndrome Study Group (IRLSSG) provides diagnostic criteria in accordance with clinical examinations and documentation for RLS [18].

The cause of RLS is still unclear; however, dopamine chemicals are believed to be involved in the development of this disorder [29]. According to Suzuki et al., not only the dopaminergic treatments may alleviate the RLS symptoms, but also migraine headaches (if treated with certain therapies such as TCA's and antiepileptic agents such as topiramate) may induce RLS [30]. Medical examinations and history are used to diagnose RLS, and if the disorder progresses, testing for anemia such as iron deficiency is required [29].

According to a study conducted by Fuh et al. the prevalence of RLS in patients with migraine in Western countries is higher than in Asian nations, and this may be related to genetics [12]. RLS, such as migraine, is a neurological disorder. In recent years, the relationship between these two disorders has been investigated in different pieces of research. These two disorders are similar in terms of prevalence in the general population [17].

van Oosterhout et al. reported that the prevalence of RLS in migraineurs is approximately 17% [25]. Also, according to another study conducted by Schürks et al. on a female population, the prevalence of RLS in migraine patients was reported to be 14.5% [23]. According to the results of our study and the survey of 15,196 patients, the overall prevalence of RLS in migraineurs is 16.9%.

The most important limitation of this study was the lack of access to the full text of some studies.

## 5. Conclusion

This study showed that RLS is observed in patients with migraine. The results obtained can be used for more focused research works aimed at determining the causes and consequences of RLS in migraine patients. In addition, this study provides a context for further studies on the treatment and impact of different therapies in the RLS population. Future studies may focus on the comparison of the effects of dopaminergic treatments in patients with RLS and migraine.

## Figures and Tables

**Table 1:** Studies included in the meta-analysis.

Study	Year published	Location	Age	Sample size	Prevalence
Calhoun et al. [15]	2006	North Carolina	39.47 ± 11.32	147	23.8
Chen et al. [16]	2010	Taipei	42.1 ± 13.3	772	11.3
Chen et al. [17]	2015	Taiwan	—	403	9.3
Cho et al. [18]	2015	Korea	—	143	9.09
Cologno et al. [19]	2008	Italy	37.1 ± 10.8	164	26
d'Onofrio et al. [20]	2011	Italy	38.2 ± 14.1	63	9.52
Muayqil et al. [21]	2018	Saudi Arabia	30.99 ± 10.76	1330	29.2
Ødegård et al. [22]	2010	Norway	—	51	19.6
Schürks et al. [23]	2012	Germany	62.5 ± 6.3	6857	14.5
Sevindik et al. [24]	2017	Turkey	11.36 ± 3.34	65	33.84
van Oosterhout et al. [25]	2016	Netherland	45.1 ± 11.6	2385	16.9
Winter et al. [26]	2013	France	67.3 ± 8.2	2816	8.7

## Data Availability

Datasets are available through the corresponding author upon reasonable request.

## Abbreviations

RLS: Restless legs syndrome
SID: Scientific Information Database
STROBE: STrengthening the Reporting of OBservational studies in Epidemiology
PRISMA: Preferred Reporting Items for Systematic Reviews and Meta-Analyses
WHO: World Health Organization
RLS/WED: Restless legs syndrome/Willis–Ekbom disorder
IRLSSG: International Restless Legs Syndrome Study Group.
